# Elevational Gradients Drive Divergent Community Assembly Processes of Different Plant Community Types on the Highest Island of Fujian, China

**DOI:** 10.3390/plants15213246

**Published:** 2026-10-22

**Authors:** Xingwei Yu, Qingyan Wen, Yaping Deng, Yulu Mo, Ruilong Wen, Yingxue Wang, Chuanyuan Deng

**Affiliations:** 1 College of Landscape Architectrue and Art, Fujian Agriculture and Forestry University, Fuzhou 350002, China; 15880266960@163.com (X.Y.); 18379890502@163.com (Q.W.); 19898864763@163.com (Y.D.); myl4793@163.com (Y.M.); wrl_469@163.com (R.W.); 2 Zhengzhou Jiajing Flower Border Horticulture Co., Ltd., Zhengzhou 450103, China; 3 Fuzhou Urban Ecosystem Research Station in Fujian, Fuzhou 350012, China

**Keywords:** dispersal limitation, environmental filtering, phylogenetic structure, forest community, grassland community, shrubland community

## Abstract

Community assembly along elevational gradients may differ among plant community types, especially on islands where environmental stress and isolation are strong. We surveyed grassland, shrubland, and forest communities in low (<300 m) and high (>300 m) elevation zones on Dayu Shan Island, Fujian Province, China, and measured leaf functional traits of the dominant herb *Miscanthus sinensis* Andersson and shrub *Mallotus japonicus* (L.f.) Müll.Arg. We evaluated how elevation drives community assembly of the three community types, using β-diversity, phylogenetic structure, and intraspecific trait variation. In shrubland communities, total β-diversity and species turnover were higher in the low-elevation zone, and geographic distance was their strongest predictor, followed by elevation. In forest communities, elevation was the only significant predictor of β-diversity and species turnover. In grassland communities, the models explained little variation and were not significant overall, although elevation was the only significant individual predictor. The abundance-difference component did not differ between elevation zones in grassland or shrubland communities. Phylogenetic structure differed among community types. Forest communities showed a significant shift from random phylogenetic structure at low elevation to phylogenetic clustering at high elevation, with soil pH as the main correlate. Shrubland communities showed a marginal shift from phylogenetic clustering at low elevation to phylogenetic overdispersion at high elevation; their net relatedness index increased with aspect (southness) and decreased with wind speed. Grassland communities did not differ significantly between elevation zones and remained predominantly phylogenetically random, with soil pH as the main environmental correlate. At high elevation zones, *M. sinensis* had thinner leaves and higher leaf nitrogen content, while *M. japonicus* had lower specific leaf area and higher leaf tissue density. Overall, different community types responded divergently to elevational change, highlighting the need for functional-group-specific conservation.

## 1. Introduction

Community assembly refers to the process through which species with different characteristics coexist in a particular habitat after environmental filtering and interspecies interactions [1]. Currently, discussions on community assembly mechanisms mainly focus on deterministic and stochastic processes. Although both processes jointly influence community assembly [2,3], the dominant process often shifts over time and space. Abiotic factors such as soil [4] and climate [5,6] can drive community assembly through environmental filtering, whereas biotic factors such as species competition [7] can shape community assembly through resource availability and competitive interactions. Elevation is an important geographic characteristic that can be used in ecological studies given its strong association with environmental conditions [8]. It typically affects the assembly indirectly by altering climatic conditions, soil properties, and other environmental factors [9,10]. In eastern North American forests, the relationship between phylogenetic relatedness and temperature was steeper along elevational gradients than along latitudinal gradients, suggesting a stronger coupling between elevation and community assembly [11]. Therefore, elevational gradients can be treated as natural laboratories in which site conditions naturally change with elevation.

High-elevation areas typically experience stronger environmental filtering, leading to a decline in plant diversity [12]. In comparison to mainland areas, islands often exhibit a lower tree line [13], suggesting that they exacerbate environmental filtering effects at higher elevations. Therefore, investigating plant community assembly along elevational gradients on islands is essential. Island vegetation plays a critical role in maintaining global biodiversity [14]. Understanding the changes in the process of plant community assembly along the elevation gradient and the potential causal factors on islands will help formulate effective conservation and management strategies for island vegetation.

Community assembly plays a crucial role in species coexistence and the maintenance of diversity [15,16]. Plant species diversity not only reflects the dynamic development and complexity of community structure but also the stability of communities, making it a key feature of community organization [17]. Beta diversity measures the rate of change in species composition along environmental gradients [18]. By decomposing beta diversity into species abundance difference and species turnover [19], we can better understand how plant communities are assembled to adapt to environmental conditions [20]. This enhances our understanding of the relationship between beta diversity and community assembly. Furthermore, the species composition of a community is shaped by both evolutionary history and ecological processes. By analyzing the phylogenetic relationships among species, we can explore the current state and underlying causes of community species composition from an evolutionary perspective [21]. However, methods that rely solely on phylogenetic structure, without considering functional traits, have limitations in explaining community assembly processes [22]. Studies have shown that the phylogenetic traits of a community cannot be equated with the functional traits of its species [23]. Functional diversity and species diversity respond to environmental factors through different ecological processes [24]. These findings highlight the need to incorporate functional traits when exploring the key drivers of plant community assembly. Additionally, Recent studies have demonstrated the significant impact of intraspecific variation on community dynamics and ecosystem functions [25,26,27]. Therefore, considering intraspecific variation is crucial for investigating community assembly mechanisms. Combining species diversity, phylogeny and functional traits can provide deeper insights into the community assembly process [28].

Plants with different life forms have distinct evolutionary histories, and their community structure [29,30] and assembly [31,32] are influenced by different environmental factors. Additionally, in different plant functional groups, the environmental factors driving species turnover differ [33]. By comparing the assembly processes of various community types (e.g., grasslands, shrublands, and forests), we can gain a deeper understanding of community assembly mechanisms and develop effective conservation and management strategies.

Fujian Province in China, a subtropical region, is home to numerous islands. Vegetation on these islands is facing unprecedented threats, driven by both climate change and human activities. Investigating the elevational patterns of plant community assembly and the underlying drivers is key to developing effective conservation and management strategies. Dayu Shan Island, the highest island in Fujian with a maximum elevation of 541.4 m, provides an ideal natural site for studying elevational patterns of plant community assembly in island mountain ecosystems. Previous research on island plant communities has largely focused on island groups [34,35] or environmental factors [36]. Recent studies have also shown that climatic variables can mediate plant–soil interactions and ecosystem functioning [37], while disturbance gradients can strongly affect herb, shrub, and tree communities by altering floristic diversity and soil physicochemical properties [38]. Such effects may be particularly pronounced on high-elevation islands, where steep environmental gradients coincide with strong climatic stress. Although elevational patterns of community assembly have been examined in some mainland and mountain systems [11,20,31,39], only a few studies have addressed such patterns on islands [34,35], and even fewer have compared different plant community types within the same island system [36].

Considering the issues described above, we designed this study to answer three specific questions on a high-elevation island: (1) How do the dominant community assembly processes shift along elevational gradients for different plant community types (grassland, shrubland, and forest), as inferred from the complementary perspectives of β-diversity, phylogenetic structure, and intraspecific functional trait variation? (2) Which environmental factors (soil and climatic variables) mediate the effects of elevation on these assembly processes, and what is the relative importance of environmental heterogeneity versus dispersal limitation in driving species turnover across each community type? (3) How do the responses of different community types to high-elevation island environments differ, particularly in terms of their phylogenetic structure and trait-based adaptive strategies? By addressing these questions, we aimed to provide an understanding of community assembly under extreme insular conditions and to inform functional-group-specific conservation approaches.

## 2. Materials and Methods

### 2.1. Study Area

**Figure 1:**
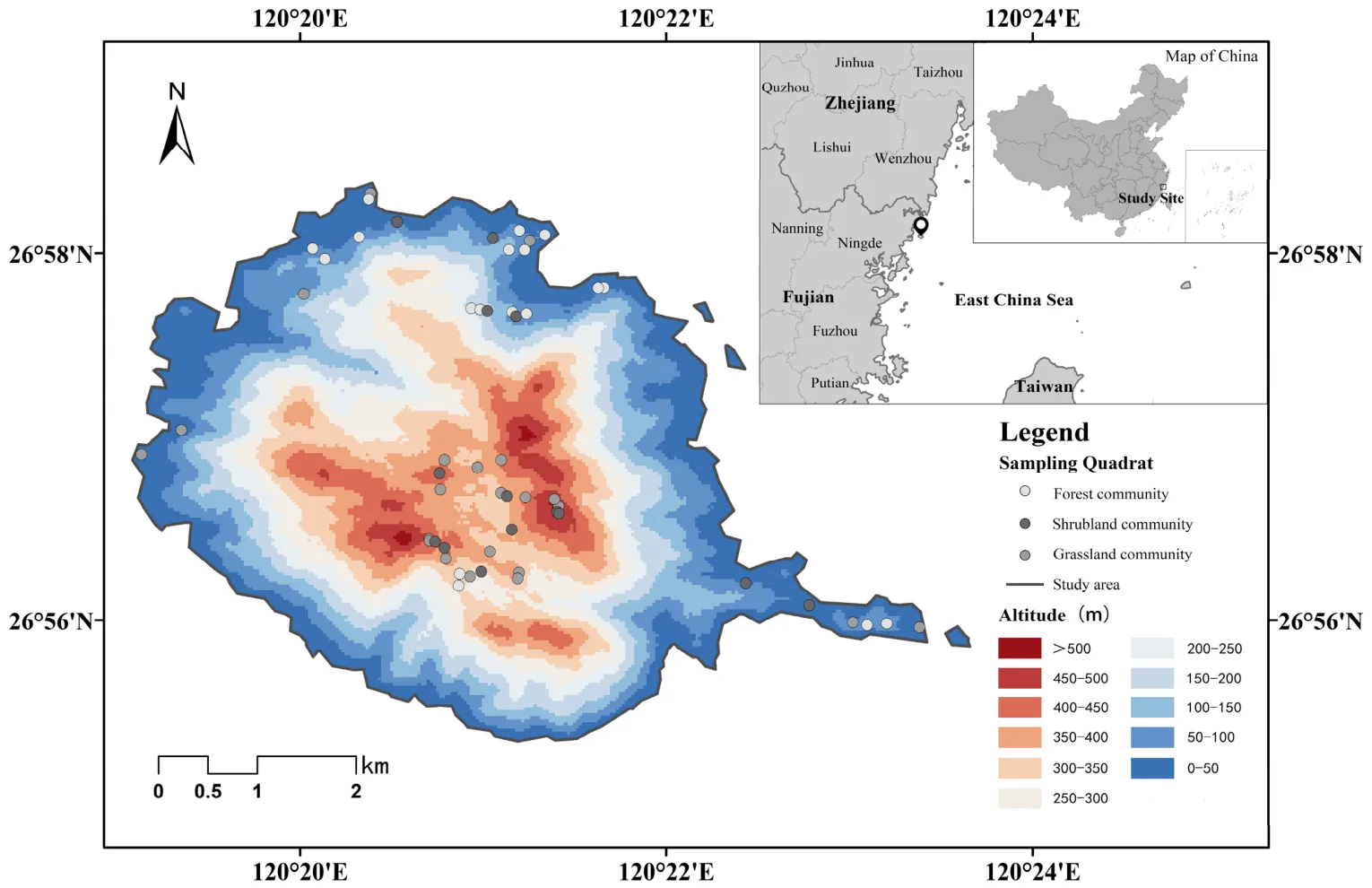
Location of the study sites and quadrats at the Dayu Shan Island.

The study was conducted on Dayu Shan Island (Figure 1), located in Fujian Province, southeastern China (120°17′ E–120°24′ E, 26°55′ N–26°58′ N). The island has a subtropical maritime monsoon climate. During winter, the average wind speed is high, and in summer, typhoons are more likely to occur. The annual average temperature is 15.2 °C, and areas at higher elevations experience an average of 5.2 frost days per year. The annual average precipitation is 2068.8 mm and annual evaporation is 1334.2 mm. The forest cover is 36.97%. Dayu Shan Island is the largest island in eastern Fujian, with an area of 21.22 km^2^. It is also the highest island in Fujian, with the highest peak, Hongjidong Mountain (541.4 m), and other prominent peaks, including Houhushanwei Mountain (405.4 m) and Tian’aogang Mountain (455.4 m), totaling more than 20 peaks.

### 2.2. Investigation of Plant Community

A field survey of plant communities was conducted during the growing season in June and July 2023 (Figure 1). The study area was categorized into two elevational zones—low-elevation (<300 m) and high-elevation (>300 m). The two zones were separated at 300 m, which lies close to the mid-point of the island’s elevational range (0–541.4 m), and sampling was stratified by these zones. This clean separation makes the contrast between the two zones unambiguous, and it does not constrain the analyses that treat elevation as a continuous variable.

In total, 54 quadrats were sampled, including 18 forest quadrats, 22 shrubland quadrats, and 14 grassland quadrats. Of these, 29 were located in the low-elevation zone (16 forest, 7 shrubland and 6 grassland quadrats) and 25 in the high-elevation zone (2 forest, 15 shrubland and 8 grassland quadrats). Each forest quadrat covered an area of 20 m × 20 m, within which 4 shrubland quadrats (5 m × 5 m) and 4 herbaceous quadrats (1 m × 1 m) were placed at the corners to survey the shrub and herb layers. Shrubland quadrats were 10 m × 10 m, with 4 herbaceous quadrats (1 m × 1 m) placed at the corners for herb layer assessment. Grassland quadrats were similarly 10 m × 10 m in size, with 4 herbaceous subquadrats (1 m × 1 m) placed at the four corners to assess the herbaceous vegetation of the entire grassland quadrat. The species composition, number, abundance, coverage, and height within the quadrats were recorded. For tree species, diameter at breast height (DBH) was also measured. All vascular plant species were identified in the field based on morphological characteristics, with reference to the iPlant database, https://www.iplant.cn/ (accessed from 25 June 2023 to 24 July 2023) When field identification was uncertain, the plants were photographed, and plant specimens were collected and transported to the laboratory for identification by taxonomic experts.

The coordinates, elevation, slope, and aspect of each quadrat were recorded. Aspect was expressed as a continuous, circular-derived variable, southness, defined as southness = −cos(θ), where θ is the aspect in radians. Southness ranges from −1 (due north), through 0 (due east or west), to +1 (due south). Higher values indicate progressively more southerly orientations, correlating with longer sunlight exposure and consequently drier, hotter habitats [40].

### 2.3. Environmental Variables

In each quadrat, five soil cores (with a diameter of 5 cm, 0–20 cm depth) were randomly collected and mixed evenly to form a composite sample. The samples were then taken to the laboratory for analysis of soil properties following the standardized protocols described in *Methods of Soil and Agro-Chemical Analysis* [41]. Soil water content (SWC) was measured by oven-drying fresh soil at 65 °C until reaching a constant weight. Soil pH was determined using a PHS-3E meter (INESA, Shanghai, China). Soil organic matter (SOM) was determined via potassium dichromate oxidation. Total nitrogen (TN) and alkaline-hydrolyzable nitrogen (AN) were determined using the Kjeldahl method. Total phosphorus (TP) and available phosphorus (AP) were determined using the molybdenum-antimony colorimetric method. Total potassium (TK) and available potassium (AK) were determined using flame photometry, and soluble salt (S) was determined using the gravimetric method.

The climate data used in this study were obtained from the WorldClim database (http://www.worldclim.org) (accessed from 1 September 2023 to 15 September 2023), which provides global meteorological data for the period 1970–2000. We used WorldClim version 2.1 [42] at a spatial resolution of 30 arc-seconds (approximately 1 km). Based on the latitude and longitude of the surveyed quadrats, the 19 standard bioclimatic variables (bio1–bio19) were extracted. Mean annual wind speed was obtained separately from the WorldClim 2.1 wind-speed layer, which is provided as 12 monthly layers (m s^−1^). These layers were averaged to obtain the annual value.

### 2.4. Selection and Determination of Functional Traits

Leaves are essential to a plant’s growth, metabolism, and ecological strategies [43]. Compared to roots and stems, leaf functional traits are not only easier to measure but also provide a better reflection of a plant’s ecological strategies [44]. The leaf functional traits selected in this study include leaf area (LA), leaf thickness (LT), specific leaf area (SLA), leaf tissue density (LTD), leaf water content (LWC), leaf nitrogen content (LNC), and leaf carbon content (LCC). LA, SLA, and LNC are key indicators of photosynthetic efficiency and resource acquisition [44,45,46]. LTD and LCC reflect a plant’s capacity to withstand environmental stresses [47,48]. LT and LWC are closely tied to its water management strategies [49,50].

Healthy and intact leaves were randomly collected from 10 to 15 individual plants of the most dominant shrub (*Mallotus japonicus* (L.f.) Müll.Arg.) and herb (*Miscanthus sinensis* Andersson) species in surveyed quadrats (Table A1). Among these, leaves of *M. sinensis* were collected from 18 quadrats, while those of *M. japonicus* were sampled from 14 quadrats. In each quadrat, ten fully expanded, healthy leaves were collected per species from different individuals at different positions. The leaves were measured for functional traits in the laboratory on the same day [51]. LT was measured with a digital caliper at three different positions (top, middle, and bottom), avoiding the veins, and the average value was calculated from three measurements. LA was determined by scanning the leaf images and analyzing them with ImageJ software (version 1.53e). After measuring fresh leaf weight (FLW), the leaves were oven-dried at 65 °C until constant weight was achieved, followed by dry leaf weight (DLW) measurement. LWC was calculated using the formula: LWC = (FLW − DLW)/DLW × 100%. SLA was calculated as: SLA = LA/DLW. LTD was calculated as: LTD = DLW/(LA × LT). LCC and LNC were determined using an elemental analyzer (varioEL).

### 2.5. Data Analysis

#### 2.5.1. β-Diversity and Its Influencing Factors

β-diversity between quadrats was quantified from a community matrix of species abundances. The dissimilarity coefficient used was the quantitative (abundance-based) form of the Sørensen dissimilarity, the percentage difference index [19], *β*_total_ = (*B* + *C*)/(2*A* + *B* + *C*), where *A* is the sum of the minimum abundances of species shared by the two quadrats, and *B* and *C* are the sums of the abundances of species occurring only in the first and only in the second quadrat, respectively. This coefficient is numerically identical to the Bray–Curtis dissimilarity. *β*_total_ and its two components were computed with the beta.div.comp function of the R package ‘adespatial’ [52], which implements the framework described by Legendre [19]. The decomposition follows the abundance-based partition of Baselga [53], which separates *β*_total_ into a replacement component (*β*_repl_) and an abundance-difference component (*β*_AD_). The two components satisfy *β*_total_ = *β*_repl_ + *β*_AD_.

β-diversity and its components were compared between the low- (<300 m) and high-elevation (>300 m) zones separately for grassland and shrubland communities; forest communities were not compared between zones because only two forest quadrats occurred in the high-elevation zone. Because the pairwise dissimilarities in a distance matrix are not independent observations, differences between zones were tested with a quadrat-level restricted permutation test. For each community type and each β-diversity component, the test statistic was the difference between the mean within-zone pairwise dissimilarity of the low-elevation quadrats and that of the high-elevation quadrats. Elevation-zone labels were randomly reassigned among quadrats while keeping the two group sizes fixed, and the statistic was recalculated for each set of permuted labels, so that the unit of permutation was the quadrat rather than the quadrat pair. Two-sided *p* values were obtained as the proportion of permuted statistics at least as extreme as the observed value. All 3003 possible label assignments were enumerated for grassland communities, and 9999 random permutations were used for shrubland communities. Analyses were performed in R 4.3.0 [54].

To further investigate the impact of geographic distance and environmental factors on β-diversity across different community types, we conducted multiple regression analysis using the multiple regression on dissimilarity matrices (MRM) approach. Before applying MRM, the redundancy of environmental variables was assessed using the varclus function from ‘Hmisc’ package [55]. Factors with Spearman’s *ρ*^2^ < 0.6 were retained (aspect, slope, S, pH, TK, AK, AP, TP, SW, SOM, mean diurnal range, precipitation seasonality), while only elevation, AN, and mean annual wind speed were kept for factors with *ρ*^2^ > 0.6. Euclidean distances were used to quantify environmental differences between quadrats, calculated using the vegdist function in ‘vegan’ package [56]. The nine soil variables (S, pH, TK, AK, AP, TP, SW, SOM, AN) were combined into a single soil factor and the three climate variables (mean diurnal range, precipitation seasonality, mean annual wind speed) into a single climate factor, each expressed as a Euclidean distance matrix among quadrats. Geographic distances between quadrats were computed using the distm function from ‘geosphere’ package [57] based on their latitude and longitude. We then implemented a matrix randomization procedure with standardized predictor variables using the MRM function in ‘ecodist’ package [58]. MRM was then fitted for each community type and each β-diversity component with six standardized predictors entered simultaneously (geographic distance, aspect, slope, elevation, soil factor and climate factor). All predictors are retained and the models are directly comparable among community types and components.

#### 2.5.2. NRI and Its Influencing Factors

The net relatedness index (NRI) was used to analyze the phylogenetic structure of the communities [59]. A phylogenetic hypothesis for the 246 species in the species list was generated in R with the package ‘V.PhyloMaker2’ [60] by binding the species list to the megaphylogeny GBOTB.extended.LCVP [61]. Genus- and family-level node information was taken from nodes.info.1.LCVP, and species absent from the megaphylogeny were placed using the default binding scenario of phylo.maker () (scenario S3). Under this scenario, a species whose genus is represented in the megaphylogeny is attached at the basal node of that genus, whereas a species whose genus is absent is inserted within the branch subtending its family. Of the 246 species, 186 matched a tip in the megaphylogeny directly, and the remaining 60 required binding. Of these, 59 were placed successfully. One species (*Grona heterocarpos*) could not be assigned to a genus or family represented in the megaphylogeny and was excluded. The final tree comprised 245 species. For each quadrat, the mean phylogenetic distance (MPD) among the species recorded in it was calculated from patristic distances on this tree. The observed MPD was compared with a null distribution generated by shuffling the tip labels of the phylogenetic distance matrix 999 times, which corresponds to the null.model = “taxa.labels” option in the R package ‘picante’ [62]. Under this null model, each quadrat is compared with a random draw of the same number of species from the species pool, here taken as all species represented in the phylogeny. NRI was calculated as the negative standardized effect size of MPD, NRI = −(MPD_obs − MPD_null)/SD (MPD_null), where MPD_obs is the observed MPD of the quadrat, and MPD_null and SD (MPD_null) are the mean and standard deviation of MPD across the 999 randomizations. MPD was calculated from species presence–absence and was not weighted by species abundance. If NRI > 0, it indicates that the average phylogenetic distance between species in the quadrat is lower than expected by chance, suggesting a clustered phylogenetic structure; if NRI < 0, it implies that the average phylogenetic distance between species is higher than expected by chance, indicating a divergent phylogenetic structure. The closer the NRI value is to 0, the more random the phylogenetic structure of the community. The *t*-test was used to examine whether the NRI of forest, shrubland, and grassland communities differed between low- (<300 m) and high- (>300 m) elevation zones.

Next, we searched for predictors of NRI of three community types using multiple regression models [30]. The best model among all models was assessed using the AICc value; that is, the lower the AICc, the better the model [63]. We used the dredge function in ‘MuMIn’ package [64]. All predictors and response variables were Z-score standardized to interpret parameters on a comparable scale. We further quantified the independent contributions of significant predictors through hierarchical partitioning analysis implemented in ‘rdacca.hp’ package [65].

Finally, we used piecewise structural equation modelling (pSEM) to examine how geographic and environmental factors influence community assembly processes, as reflected by NRI in the three community types. The geographic and environmental factors were included. The geographic factors were elevation and slope aspect. The environmental factors were the soil and climatic variables retained in the AICc-best multiple regression model of NRI for that community type (Section 3.2). soil pH for forest, mean annual wind speed for shrubland, and soil pH and precipitation seasonality for grassland. The hypothesised causal structure was: geographic factors → environmental factors → NRI. Because aspect was retained as a direct predictor of NRI in the AICc-best model for shrubland, the path aspect → NRI was additionally included in that model. All equations were linear models fitted to plot-level values, so the unit of observation was the quadrat (*n* = 14, 22 and 18 for grassland, shrubland and forest, respectively). Because sample sizes were small, model adequacy was assessed with Fisher’s C, for which *p* > 0.05 indicates that a model is consistent with the data. All analyses were carried out in R 4.3.0 [54] using the ‘piecewiseSEM’ package [66].

#### 2.5.3. The Effect of Elevation on Leaf Functional Traits

To compare leaf functional traits of the dominant herb (*M. sinensis*) and shrub (*M. japonicus*) species between low- (<300 m) and high- (>300 m) elevation zones, we first assessed normality and homogeneity of variances for each trait. When both assumptions were met, independent-samples *t*-tests were used; otherwise, Mann–Whitney U tests were applied.

## 3. Results

### 3.1. Changes in β-Diversity of Different Plant Community Types Along Elevational Gradients and Their Driving Factors

**Figure 2:**
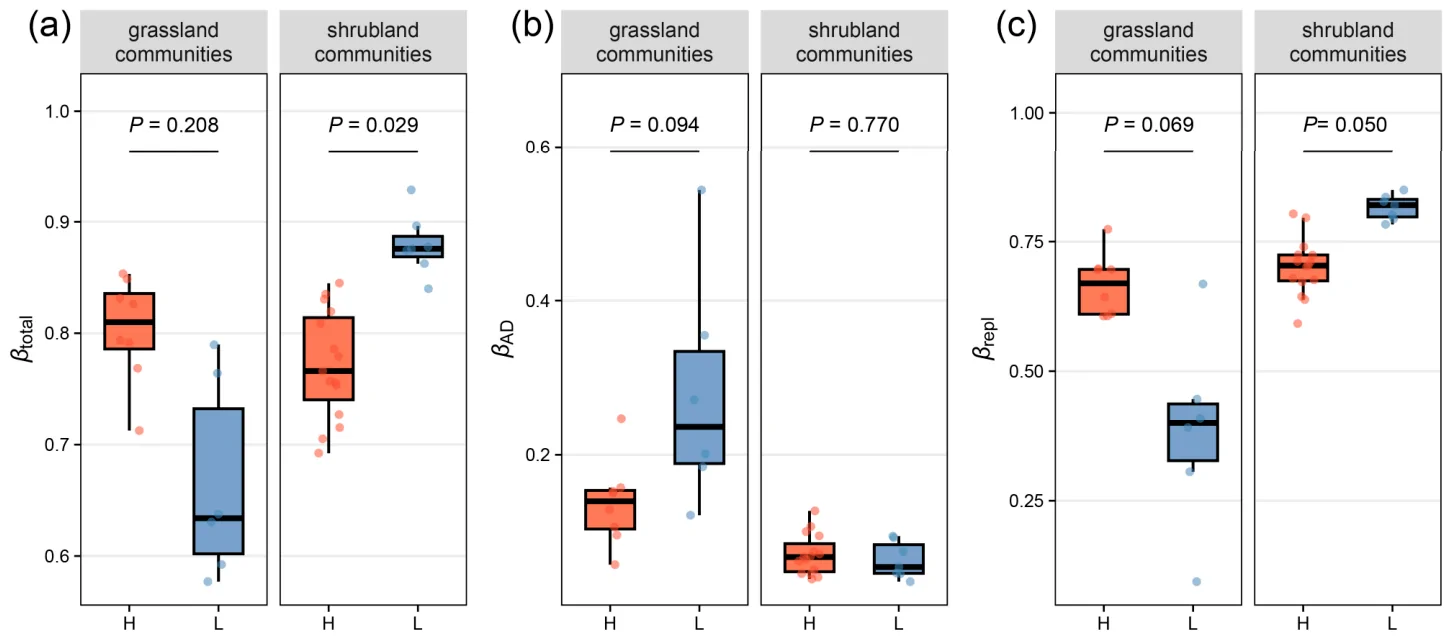
β-diversity and its components between low-elevation (L, <300 m) and high-elevation (H, >300 m) zones in grassland and shrubland communities. (**a**) Total β-diversity (*β*_total_), (**b**) species abundance difference (*β*_AD_) and (**c**) species turnover (*β*_repl_). Forest communities were not compared between zones because only two forest quadrats occurred above 300 m. *p* values are from quadrat-level restricted permutation tests (grassland, *n* = 6 vs. 8 quadrats; shrubland, *n* = 7 vs. 15).

In both high- and low-elevation zones, species turnover was the dominant contributor to total β-diversity in forest, shrubland, and grassland communities (Table A2). Total β-diversity differed significantly between the two elevation zones in shrubland communities (*p* = 0.029) but not in grassland communities (*p* = 0.208; Figure 2a). For the components of β-diversity, species turnover in shrubland communities was higher in the low-elevation zone (*p* = 0.050), whereas grassland communities showed the opposite trend, which was not statistically significant (*p* = 0.069; Figure 2c). The species abundance difference component did not differ between elevation zones in either community type (grassland, *p* = 0.094; shrubland, *p* = 0.770; Figure 2b).

MRM was used to determine the relative contributions of environmental factors, geographic distance, and elevation to β-diversity and its components of different community types (Table 1). For forest communities, the MRM model explained 35.0% and 28.8% of the variability in β-diversity and species turnover, respectively (*p* = 0.007 and *p* = 0.022), with elevation being the sole significant predictor (*b* = 0.379 and 0.550, *p* < 0.05 and *p* < 0.01, respectively). For grassland communities, the MRM model explained less than 20% of the variability in β-diversity and species turnover and was not significant overall (*R*^2^ = 0.086 and 0.127, *p* = 0.555 and 0.253, respectively); elevation was again the only significant predictor (*b* = 0.294 and 0.336, *p* < 0.05). For shrubland communities, the MRM model explained 29.0% and 29.4% of the variability in β-diversity and species turnover (*p* < 0.001), respectively. Geographic distance contributed the largest partial regression coefficients (*b* = 0.537 and 0.493, *p* < 0.001), while elevation contributed smaller but significant coefficients (*b* = 0.165 and 0.218, *p* < 0.05 and *p* < 0.01, respectively).

### 3.2. Changes in Community Assembly of Different Plant Community Types Along Elevational Gradients and Their Influencing Factors

**Figure 3:**
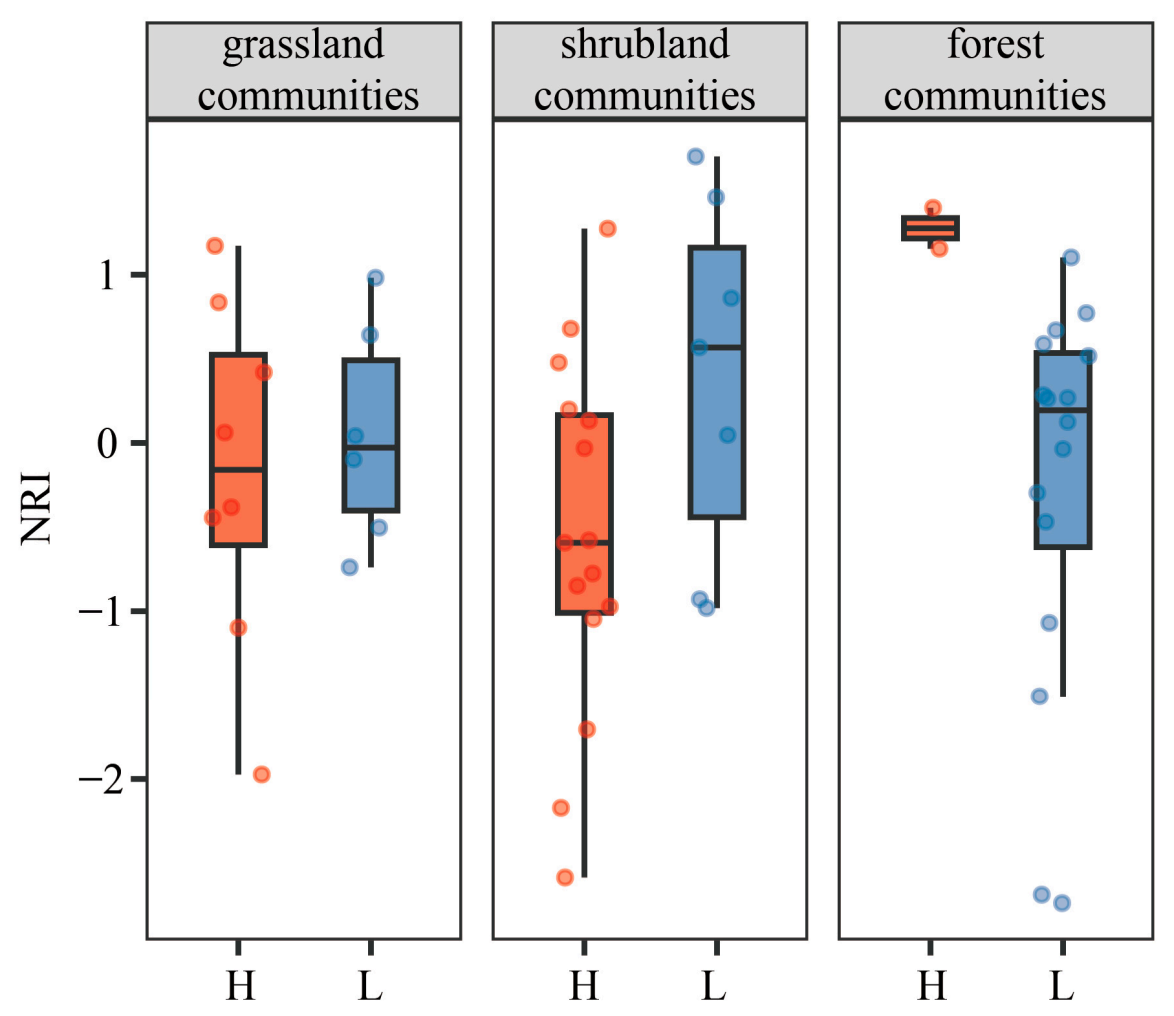
The net relatedness index (NRI) differences between two elevation zones across three community types. H: high elevation zone (>300 m), L: low elevation zone (<300 m).

Along the elevational gradients (Figure 3), the phylogenetic structure of grassland communities did not differ between the two elevation zones (*p* = 0.62). For shrubland communities, the phylogenetic structure did not differ significantly between elevation zones (*p* = 0.07), although there was a marginal tendency towards overdispersion at high elevation. In contrast, the phylogenetic structure of forest communities differed significantly between zones (*p* < 0.001).

**Figure 4:**
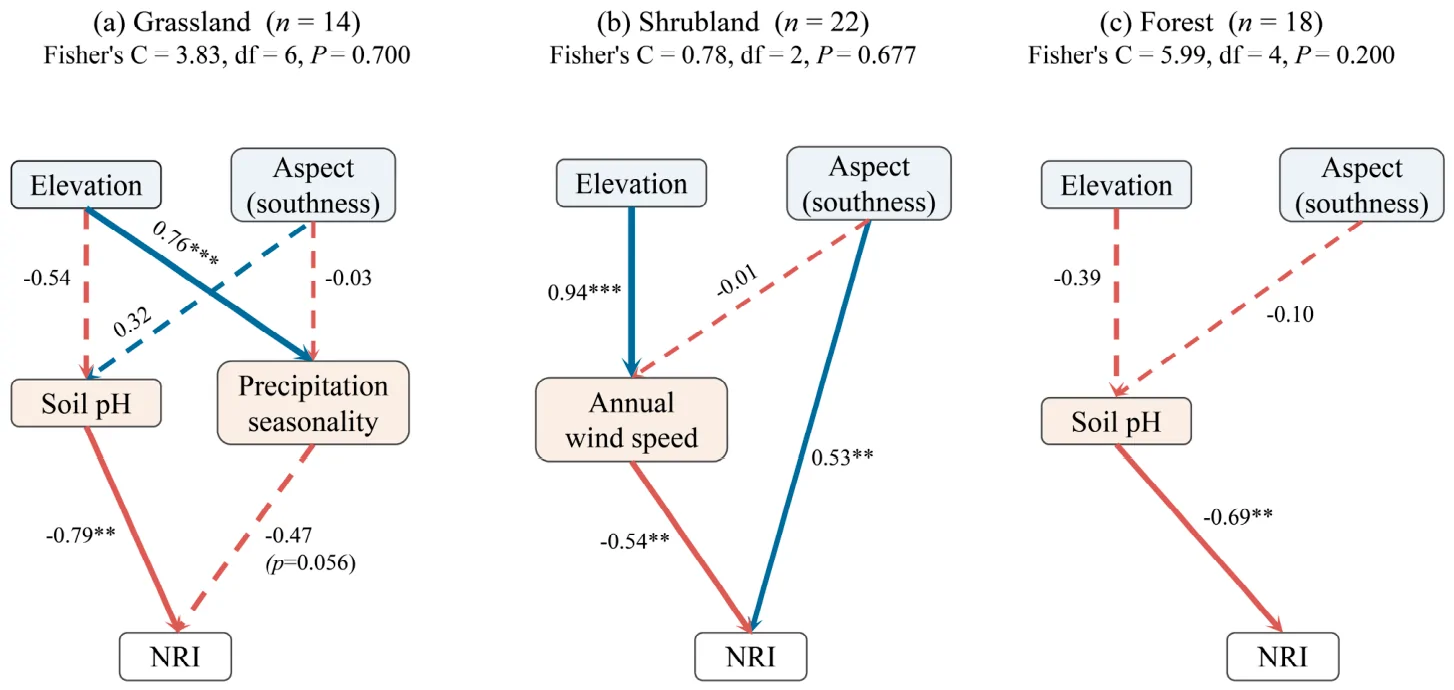
The direct and indirect impacts of factors on the net relatedness index (NRI) across three plant community types. (**a**) grassland communities, (**b**) shrubland communities, (**c**) forest communities. Numbers adjacent to the arrows are standardized path coefficients together with the corresponding *p* values. *** *p* < 0.001, ** *p* < 0.01. Solid arrows indicate *p* < 0.05 and dashed arrows *p* ≥ 0.05. Blue and red indicate positive and negative effects, respectively. Fisher’s C and the d-separation tests of all independence claims implied by the model are given above each panel.

For grassland communities, NRI decreased with soil pH (independent contribution 41.2%, *p* = 0.005), whereas precipitation seasonality was retained in the model but did not reach statistical significance (5.2%, *p* = 0.056; Table 2, Figure 4a). Elevation was not significantly related to soil pH, but had a strong positive effect on precipitation seasonality (*b* = 0.76, *p* = 0.007; Figure 4a). In shrubland communities, NRI increased with aspect (southness) (22.5%, *p* = 0.004, Table 2; Figure 4b) and decreased with mean annual wind speed (23.5%, *p* = 0.004, Table 2; Figure 4b). Elevation was strongly positively associated with wind speed (*b* = 0.94, *p* < 0.001, Figure 4b), and wind speed in turn was negatively related to NRI (*b* = −0.54, *p* = 0.004, Table 2; Figure 4b). For forest communities, soil pH was the only predictor retained in the model (independent contribution 44.3%, *b* = −0.69, *p* = 0.002; Table 2, Figure 4c). Elevation was not significantly related to soil pH (*b* = −0.39, *p* = 0.124; Figure 4c).

### 3.3. Changes in Leaf Functional Traits of Island Plants Along Elevational Gradients

We compared the changes in leaf functional traits of *M. sinensis* and *M. japonicus* along elevational gradients (Table 3). The results showed that, in the high-elevation zone, *M. sinensis* exhibits smaller leaf thickness (*p* < 0.05) and higher leaf nitrogen content (*p* < 0.05), while *M. japonicus* shows smaller leaf area (*p* < 0.01), lower specific leaf area (*p* < 0.05), and higher leaf tissue density (*p* < 0.05).

## 4. Discussion

### 4.1. Effect of Elevation on Species Turnover of Different Plant Community Types

Our results showed that shrubland communities had significantly higher species turnover in the low-elevation zone than in the high-elevation zone, whereas grassland communities showed a non-significant tendency toward higher turnover in the high-elevation zone (Figure 2). This indicates that the variation in species turnover is largely explained by elevational differences, which aligns with previous studies on montane island vegetation [67]. Furthermore, studies have shown that species turnover tends to be higher under extreme environmental conditions [68]. However, the pattern differed between community types: shrubland communities showed significantly lower species turnover in the high-elevation zone, whereas grassland communities showed only a non-significant tendency toward higher turnover at high elevation (Figure 2c). Some research also suggests that human management activities can reduce species turnover in grassland communities [69]. In our study area, the low-elevation zone tends to experience more human disturbance. During field surveys, we observed that some Miscanthus sinensis-dominated patches had been cleared and replanted with shrubs or trees, likely through local vegetation management. This may partly explain the non-significant tendency toward lower species turnover in low-elevation grassland communities (Figure 2), although the timing, extent, and intensity of these activities could not be quantified. However, due to the difficulty in quantifying such disturbances, further investigation is needed to fully understand their impact. Interestingly, shrubland communities had significantly lower species turnover in the high-elevation zone, which contrasted with the non-significant trend observed in grassland communities (Figure 2). This may be because species turnover in shrubland communities was strongly affected by dispersal limitation (Table 1). In the high-elevation zone, reduced geographic distances and stronger winds may alleviate dispersal constraints, facilitating increased species exchange between shrubland communities, thereby reducing species turnover. Previous studies have shown that species turnover in different plant functional groups is driven by different factors [33], which is consistent with our speculation and findings (Table 1). This may explain why species turnover in shrubland communities responds differently to elevation compared to grassland communities. Nonetheless, the species abundance difference component (*β*_AD_) did not change significantly along the elevational gradients in either grassland or shrubland communities (Figure 2), suggesting that different community types may maintain species diversity in response to the environmental gradient through distinct mechanisms. Although our study could not statistically compare tree community turnover between two elevation zones due to their rarity in the high-elevation zone, previous studies suggest that elevational gradients significantly influence tree mortality [70] and species turnover [71]. Notably, forests under extreme environmental conditions often exhibit higher turnover rates [68], implying that if tree communities were present in sufficient numbers at high elevation on islands, their β-diversity might follow a similar pattern driven by intensified environmental filtering. Future studies on high-elevation tree communities could test whether island tree communities conform to this pattern.

Our findings also indicate that in shrubland communities, dispersal limitation exerts an influence on turnover that is comparable to—or greater than—that of environmental heterogeneity. This aligns with existing evidence that dispersal limitation plays a critical role in shaping shrubland community assembly, even for species with high intrinsic dispersal capacity [72]. This contrast implies that the dominant assembly processes in shrubland communities differ from those operating in grassland and forest communities. However, our analysis cannot determine the underlying causes of these differences. Future research focused on the composition of different community types and the dispersal abilities of their constituent species will help clarify the mechanisms by which dispersal limitation affects species turnover [73].

### 4.2. Effect of Elevation on Dominant Community Assembly Processes of Different Plant Community Types

Regardless of elevation, the assembly of grassland communities is primarily driven by stochastic processes (Figure 3). This result is consistent with previous studies on the phylogenetic structure of herbaceous plants along elevation gradients [39]. Similarly, we found that the phylogenetic structures of forest communities in the low-elevation zone were random (Figure 3). Random phylogenetic structures may result from the effects of either neutral processes or opposing non-neutral processes [74]. In our study area, grassland communities are dominated by *M. sinensis* and *Imperata cylindrica*, which are mainly wind-dispersed and exhibit weak dispersal limitation. These species are capable of adapting to a wide range of environmental conditions. Therefore, we hypothesize that grassland communities were primarily structured by neutral processes in the altitudinally variable environment of Dayu Shan Island. In Fangshuai’s study, phylogenetic structures of woody plants in forests exhibited random patterns at larger spatial scales, attributed to the counteracting effects of opposing non-neutral assembly processes [75]. Accordingly, we propose that forest communities in the low-elevation zone are primarily structured by a combination of environmental filtering and limiting similarity. In the high-elevation zone, where tree survival and growth are constrained by limited resources, the forest communities are dominated by dwarf *Acacia confusa*, reflecting the predominance of environmental filtering. For shrubland communities, our results show that the dominant assembly process shifts from environmental filtering to limiting similarity with increasing elevation (Figure 3). This transition is closely related to resource availability. According to previous studies, when resources are abundant or coexisting organisms can effectively share them, niche overlap tends to increase [76,77,78]. The effect of resource availability on limiting similarity can vary along abiotic gradients [79]. These findings suggest that elevation has a profound influence on the assembly of shrubland communities through its impact on resource availability and environmental factors (e.g., annual wind speed and slope aspect–related conditions).

Our results indicate that the impact of elevation changes on the assembly of different community types varies, likely due to differences in the dominant environmental factors influencing each community type’s assembly (Table 2, Figure 4). In our study, soil pH significantly influenced the dominant processes shaping the assembly of grassland communities. In shrubland communities, community assembly was affected by slope aspect and annual wind speed, whereas in forest communities it was primarily influenced by soil pH. Slope aspect may indirectly influence community assembly processes by modulating a range of climatic conditions, particularly in shrubland communities (Figure 4). On Dayu Shan Island, the prevailing winds are from the southeast in summer and from the north in winter and spring. The island’s topography is steeper on the south-facing slopes and gentler on the north-facing slopes, making plant communities on the south slope more exposed to prevailing winds. Additionally, since the south-facing slope is the sunny side, it receives more solar radiation, resulting in drier habitats and thus stronger environmental filtering. Therefore, in shrubland communities, the more southward the slope aspect, the greater the environmental filtering (Figure 4). Notably, the assembly of forest communities is also affected by soil pH, which we speculate is due to the environmental filtering effect of soil conditions on tree seedlings, thereby influencing the development of forest communities [80]. However, environmental factors (e.g., AN, TP) that significantly influence species turnover do not necessarily affect NRI, suggesting that different environmental variables may play distinct roles in shaping community assembly.

We also analyzed whether herbaceous and shrub plants are subject to environmental filtering along elevational gradients from the perspective of leaf functional traits (Table 3). The variation in *M. sinensis* and *M. japonicus* in leaf traits in different elevation zones suggests that plants adapt to high-elevation environments through intraspecific variation in leaf functional traits. This may provide functional trait–based evidence that elevation exerts environmental filtering on grassland and shrubland communities, with different species adopting different adaptive strategies. In our study, *M. sinensis* exhibits thinner leaves in the high-elevation zone. This may be related to the windy conditions on the island, as thinner leaves are more flexible and less prone to damage in strong winds. Although some studies have shown that high elevation can lead to increased leaf thickness as a means of protecting metabolically active tissues such as mesophyll [81,82]. Higher leaf nitrogen content is associated with rapid plant growth [83]. *M. sinensis* exhibited higher leaf nitrogen content in the high-elevation zone, indicating that it consistently invests more resources in growth at high elevations, suggesting that the influence of elevation on *M. sinensis* on Dayu Shan Island is limited. In contrast, the high-elevation environment was associated with smaller specific leaf area and leaf area in M. japonicus, potentially limiting its ability to acquire and utilize resources. However, *M. japonicus* compensates by increasing leaf tissue density to withstand the harsh conditions in the high-elevation zone, as observed in other island plant species [49]. In this way, *M. japonicus* reallocates resources from growth and reproduction to structural tissues to cope with environmental stress at high elevations. Therefore, the high-elevation environment limits the productivity of *M. japonicus* to some extent, indicating a degree of environmental filtering. Our results further suggest that different species adopt different strategies in response to environmental change. This variation in adaptive strategies may contribute to the coexistence of different plant species.

## Figures and Tables

**Table 1:** Results of the multiple regression analysis on dissimilarity matrices (MRM) for β-diversity and its components across grassland, shrubland and forest communities. The variation (*R*^2^) of β-diversity (*β*_total_) and its components (*β*_AD_, *β*_repl_) that is explained by the remaining variables. The partial regression coefficients (*b*) and associated *p*-values of the final model are reported from permutation tests (nperm = 9999) if their significance level is <0.05. * *p* < 0.05, ** *p* < 0.01, *** *p* <0.001. Abbreviations: *β*_total_, total β-diversity; *β*_repl_, species turnover; *β*_AD_, species abundance difference.

Community Types	Component	*R* ^2^	*p*	Geographic Distance	Aspect (Southness)	Slope	Elevation	Soil Factor	Climate Factor
Grassland communities	*β*_total_	0.086	0.555	−0.191	0.048	0.014	0.294 *	0.041	0.014
	*β*_AD_	0.179	0.114	−0.108	0.275 *	0.025	−0.269 *	−0.121	−0.061
	*β*_repl_	0.127	0.253	−0.054	−0.131	−0.005	0.336 *	0.096	0.044
Shrubland communities	*β*_total_	0.290	<0.001	0.537 ***	−0.018	−0.028	0.165 *	0.057	−0.178
	*β*_AD_	0.081	0.161	−0.139	0.017	−0.000	−0.201 *	−0.105	0.127
	*β*_repl_	0.294	<0.001	0.493 ***	−0.022	−0.023	0.218 **	0.090	−0.198
Forest communities	*β*_total_	0.350	0.007	0.266	0.039	0.050	0.379 *	0.060	0.158
	*β*_AD_	0.458	<0.001	0.270	0.010	−0.011	−0.461 **	−0.031	0.412 *
	*β*_repl_	0.288	0.022	0.080	0.027	0.047	0.550 **	0.065	−0.083

**Table 2:** The best models explaining the net relatedness index (NRI) across three community types, selected by AICc. Standardized coefficients indicate the relative contribution of each predictor, while independent contributions (derived from hierarchical partitioning) reflect the proportion of variance explained solely by each variable.

Community Types	Predictors	Std. Coefficient	*p*-Value	Independent Contribution (%)
Forest communities	Soil pH	−0.69	0.002	44.3%
Shrubland communities	Aspect (southness)	0.53	0.004	22.5%
	Annual wind speed	−0.54	0.004	23.5%
Grassland communities	Soil pH	−0.79	0.005	41.2%
	Precipitation seasonality	−0.47	0.056	5.2%

**Table 3:** Variation in leaf functional traits of *M. sinensis* and *M. japonicus* between low- (<300 m) and high- (>300 m) elevation zones. Values are means ± standard deviation (SD). Asterisks (*) indicate a significant difference between low- and high-elevation zones for the respective species (*p* < 0.05).

	*M*. *sinensis*		*M*. *japonicus*	
Leaf Functional Traits	Low-Elevation Zone (<300 m)	High-Elevation Zone (>300 m)	Low-Elevation Zone (<300 m)	High-Elevation Zone (>300 m)
leaf area (cm^2^)	228.10 ± 98.62	178.79 ± 71.11	139.04 ± 44.74	82.29 ± 23.95
leaf thickness (mm)	0.17 ± 0.02 *	0.15 ± 0.01 *	0.19 ± 0.03	0.19 ± 0.02
leaf water content (%)	1.50 ± 0.52	1.72 ± 0.22	2.64 ± 1.97	1.78 ± 0.37
specific leaf area (cm^2^/g)	96.74 ± 20.77	101.74 ± 16.61	198.60 ± 66.83 *	139.86 ± 25.59 *
leaf tissue density (g/cm^3^)	0.63 ± 0.20	0.66 ± 0.12	0.29 ± 0.07 *	0.39 ± 0.06 *
leaf nitrogen content (%)	1.07 ± 0.16 *	1.27 ± 0.14 *	2.29 ± 0.33	2.54 ± 0.18
leaf carbon content (%)	46.20 ± 0.73	46.13 ± 0.44	45.98 ± 0.33	46.43 ± 0.77

## Data Availability

The data of this study are available on request.

## Appendix A

### Vegetation Overview of the Surveyed Quadrats on Dayu Shan Island

The importance value (IV) was used to quantify the status and dominance of each species in the Dayu Shan Island. It was calculated as follows:

IV_T_ = (*R*_h_ + *R*_c_ + *R*_a_)/300

IV_H_ = (*R*_h_ + *R*_c_)/200

where the IV_T_ is the species importance value of tree layer and shrub layer, the IV_H_ is the species importance value of the herb layer, the *R*_h_ is the relative height (*R*_h_ = total height of a certain plant species × 100/total height of all plant species), the *R*_c_ is the relative cover (*R*_c_ = total cover of a certain plant species × 100/total cover of all plant species), the *R*_a_ is the relative abundance (*R*_a_ = total abundance of a certain plant species × 100/total abundance of all plant species).

We recorded 264 vascular plant species, belonging to 186 genera and 87 families. The tree layer comprised only 12 species from 12 genera across 11 families. For each layer, we ranked recorded species by their importance values and identified the top 10 dominant species in the tree, shrub, and herbaceous layers (Table A1). The species with the highest importance values in each layer were *Acacia confusa* Merr. (6.025) in the tree layer, *Mallotus japonicus* (L.f.) Müll.Arg. (5.204) in the shrub layer, and *Miscanthus sinensis* Andersson (19.372) in the herbaceous layer.

**Table A1:** Dominant species in the tree, shrub, and herbaceous layers, showing the top 10 species ranked by importance value (IV, %).

Herbaceous Layer	IV (%)	Shrub Layer	IV (%)	Tree Layer	IV (%)
*Miscanthus sinensis* Andersson	19.37	*Mallotus japonicus* (L.f.) Müll.Arg.	5.20	*Acacia confusa* Merr.	6.03
*Imperata cylindrica* (L.) Raeusch.	4.92	*Eurya chinensis* R.Br.	3.87	*Camphora officinarum* Nees	5.68
*Alpinia zerumbet* (Pers.) B.L.Burtt & R.M.Sm.	3.58	*Ficus erecta* Thunb.	3.48	*Pinus massoniana* Lamb.	2.92
*Dicranopteris pedata* (Houtt.) Nakaike	3.34	*Smilax china* L.	3.42	*Casuarina equisetifolia* L.	2.26
*Aster ageratoides* Turcz.	2.22	*Rubus parvifolius* L.	2.90	*Celtis sinensis* Pers.	1.04
*Chrysanthemum indicum* L.	2.10	*Glochidion obovatum* Siebold & Zucc.	2.64	*Cunninghamia lanceolata* (Lamb.) Hook.	0.69
*Pteridium aquilinum* var*. latiusculum* (Desv.) Underw. ex A.Heller	1.76	*Rubus buergeri* Miq.	2.51	*Vernicia fordii* (Hemsl.) Airy Shaw	0.40
*Lygodium japonicum* (Thunb.) Sw.	1.51	*Heptapleurum heptaphyllum* (L.) Y.F.Deng	2.15	*Trachycarpus fortunei* (Hook.) H.Wendl.	0.13
*Erigeron canadensis* L.	1.30	*Litsea rotundifolia* var*. oblongifolia* (Nees) C.K.Allen	1.76	*Liquidambar formosana* Hance	0.09
*Dianella ensifolia* (L.) DC.	1.25	*Pueraria montana* var*. lobata* (Willd.) Maesen & S.M.Almeida ex Sanjappa & Predeep	1.50	*Archidendron lucidum* (Benth.) I.C.Nielsen	0.06

**Table A2:** Decomposition of β-diversity (*β*_total_: total β-diversity, *β*_repl_: species turnover, *β*_AD_: species abundance difference) for grassland, shrubland, and forest communities in low- (<300 m) and high-elevation (>300 m) zones. Proportions of each component to *β*_total_ are shown in parentheses.

	Grassland Communities		Shrubland Communities		Forest Communities	
	Low-Elevation Zone (<300 m)	High-Elevation Zone (>300 m)	Low-Elevation Zone (<300 m)	High-Elevation Zone (>300 m)	Low-Elevation Zone (<300 m)	High-Elevation Zone (>300 m)
*β*_total_	0.33	0.40	0.44	0.39	0.37	0.26
*β*_AD_	0.14 (42.02%)	0.07 (17.04%)	0.03 (7.20%)	0.04 (9.14%)	0.04 (10.74%)	0.06 (22.82%)
*β*_repl_	0.19 (57.98%)	0.33 (82.96%)	0.41 (92.80%)	0.35 (90.86%)	0.33 (89.26%)	0.20 (77.18%)
